# Supplementary material for: Host-inherent variability influences the transcriptional response of Staphylococcus aureus during in vivo infection
Source: Nat Commun. 2017 Feb 3;8:14268. doi: 10.1038/ncomms14268 (PMC5296661; doi:10.1038/ncomms14268)
Supplement: Supplementary Information — Supplementary Tables and Supplementary Figures [file ncomms14268-s1.pdf]

Supplementary Table 1 PERMANOVA analysis of differences in gene expression between samples from A/J and C57BL/6 mice

Main-test						
				**Test-statistic**	**p-values**	
**Source**	**df[a]**	**SS[b]**	**MS[c]**	**pseudo-F[e]**	**Permutations**	**Monte Carlo[d]**
Samples	3	1.9425E12	6.4749E11	24.804	0.0001	0.0001
Residual	8	2.0884E11	2.6104E10			
Total	11	2.1513E12				

Pair-wise test			
**Pair-wise comparisons**	**test-statistic (t)[e]**	**p-value[d]**	**Average distance between samples[f]**
Uninfected A/J vs Uninfected C57BL/6	1.316	0.1959	2.7141E5
Uninfected A/J vs Infected A/J	6.1476	0.0005	8.3287E5
Uninfected C57BL/6 vs Infected C57BL/6	4.756	0.0015	6.5137E5
Infected A/J vs Infected C57BL/6	5.2082	0.0014	6.4454E5

[a] degrees of freedom
[b] Sum of Squares
[c] Mean of Squares
[d] Monte Carlo *p*-values were generated (using 9999 Monte Carlo samples) from the asymptotic permutation distribution
[e] The t-statistic was generated using either the main test or the pair-wise test (*a posteriori*) within the PERMANOVA routine using PRIMER v. 6
[f] The average distance between pairs of conditions was calculated using the Euclidean distance algorithm

Supplementary Table 2 PERMANOVA analysis of differences in gene expression between *S. aureus* infecting A/J and C57BL/6 mice

Main-test						
				**Test-statistic**	**p-values**	
**Source**	**df[a]**	**SS[b]**	**MS[c]**	**pseudo-F[e]**	**Permutations**	**Monte Carlo[d]**
Samples	1	3,21E6	3,21E6	4,9673	0.0994	0.0262
Residual	4	2,58E6	6,46E5			
Total	5	5,79E6				

[a] degrees of freedom
[b] Sum of Squares
[c] Mean of Squares
[d] Monte Carlo *p*-values were generated (using 9999 Monte Carlo samples) from the asymptotic permutation distribution
[e] The t-statistic was generated using either the main test within the PERMANOVA routine using PRIMER v. 6

Supplementary Table 3 Primers used for qRT-PCR

Target	Forward /Reverse	Sequence (5'-3')	Annealing temperature (°C)
16S ribosomal RNA	for<br>rev	CGGTCCAGACTCCTACGGGAGGCAGCA<br>GCGTGGACTACCAGGGTATCTAATCC	70
Accessory gene regulator A (*agrA*)	for<br>rev	AACTGCACATACACGCTTACA<br>GGCAATGAGTCTGTGAGATTT	60
Alpha-hemolysin (*hla*)	for<br>rev	GGCCTTATTGGTGCAAATGT<br>AGCGAAGTCTGGTGAAAACC	60
Apolipoprotein B (*Apob*)	for<br>rev	GAAGCGCCACCAAGATCAAC<br>CAGCTTGAGTTCGTACCTGGA	60
Beta-actin (*Actp*)	for<br>rev	TGGAATCCTGTGGCATCCATGAAAC<br>TAAAACGCAGCTCAGTAACAGTCCG	60
D-serine/D-Alanine/glycine transporter (*aapA*)	for<br>rev	CGGCATATTCGCTAAAGGTGC<br>GATAACCGCTAACGCCCCAA	60
Glutamate synthase subunit beta (*gltD*)	for<br>rev	CTGGTGATACAGGGGCAGAC<br>TGCACGTGGTTCCTTACCAA	60
Glutamyl endopeptidase (*sspA*)	for<br>rev	GTTCGTCTGGGTTGTTAGGGT<br>GGAATTCATTTGGGGCGGT	60
Protein VraX (*vraX*)	for<br>rev	TTTATCGACAGTATCACCATGA<br>TCATATGATCTATATCGTCTTGTA	60
Regulatory RNAIII (RNAIIII/*hld*)	for<br>rev	TAGCACTGAGTCCAAGGAAACT<br>AGGAGTGATTTCAATGGCACAAG	60
Transcriptional regulator SarR (*sarR*)	for<br>rev	TTTAGTCAACGCAACATTTCAAGT<br>ACTGTTCTTTCGTCTTGTAAACTTC	60
Zinc metalloproteinase aureolysin (*aur*)	for<br>rev	AGCGTCTCCCTCTTTTCCAG<br>GCATGGATCGGTGACAAAAT	60

Supplementary Table 4 DEGs between *S. aureus* infecting A/J and C57BL/6 mice with greater transcript abundance during infection of A/J mice

Locus tag	Gene symbol	Description	Mean TPM[a] in A/J mice	Mean TPM[a] in C57BL/6 mice	Fold change	prob[b]	FDR[c]
SAOUHSC_00845		Hypothetical	130486,7	66410,36	1,95	1	<1,00E-15
SAOUHSC_02853		Hypothetical	36020,86	3700,52	9,44	1	2,00E-15
SAOUHSC_00371	*yflT*	Hypothetical	5693,38	2736,38	2,07	0,997066435	2,93E-03
SAOUHSC_02964	*arcR*	Hypothetical	787,21	263,57	2,95	0,99444414	5,56E-03
SAOUHSC_01477		Hypothetical	6780,60	3628,87	1,84	0,988480839	1,15E-02
SAOUHSC_01969	*gvpP*	Hypothetical	1729,37	594,54	2,87	0,980445174	1,96E-02
SAOUHSC_00101	*drm*	Phosphopentomutase	1079,18	600,27	1,78	0,977996915	2,20E-02
SAOUHSC_01181	*xynA*	Hypothetical	5594,05	1982,24	2,71	0,977767116	2,22E-02
SAOUHSC_02967	*arcD*	Arginine/ornithine antiporter	371,89	132,03	2,77	0,974020986	2,60E-02
SAOUHSC_01191	*rpmB*	50S ribosomal protein L28	19639,47	8597,22	2,21	0,972792216	2,72E-02
SAOUHSC_00686		Hypothetical	4342,19	1731,44	2,48	0,971625826	2,84E-02
SAOUHSC_01803	*aapA*	Hypothetical	431,21	196,35	2,16	0,971576556	2,84E-02
SAOUHSC_02862	*clpL*	ATP-dependent Clp protease, ATP-binding subunit ClpC	884,79	437,50	1,99	0,9704602	2,95E-02
SAOUHSC_01403	*cspA*	Cold shock protein	3629,95	1482,13	2,42	0,969955816	3,00E-02
SAOUHSC_02850	*cidB*	Hypothetical	1088,77	438,58	2,45	0,968994135	3,10E-02
SAOUHSC_01002	*qoxB*	quinol oxidase AA3 subunit II	3569,46	1518,99	2,28	0,968866128	3,11E-02
SAOUHSC_01024	*graF*	Hypothetical	8582,01	2646,52	3,18	0,968787341	3,12E-02
SAOUHSC_02702		Hypothetical	10868,04	3204,10	3,27	0,968073025	3,19E-02
SAOUHSC_02697	*tcyC*	Amino acid ABC transporter ATP-binding protein	551,76	190,95	2,84	0,965357298	3,46E-02
SAOUHSC_02665		Hypothetical	2396,27	926,44	2,54	0,961738206	3,83E-02

[a] Transcripts Per Kilobase Million; [b] Probability of differential expression; [c] False discovery rate

Supplementary Table 5 DEGs between *S. aureus* infecting A/J and C57BL/6 mice with greater transcript abundance during infection of C57BL/6

Locus tag	Gene symbol	Description	Mean TPM[a] in A/J mice	Mean TPM[a] in C57BL/6 mice	Fold change	Prob[b]	FDR[c]
SAOUHSC_02260	*hld*	Delta-hemolysin	42693,19	121705,43	-2,85	1	1,10E-14
SAOUHSC_00411.1	*psma1*	Alpha phenol soluble modulin	8810,56	33940,55	-3,89	1	3,76E-10
SAOUHSC_02566	*sarR*	Hypothetical	896,41	3189,00	-3,53	0,980309226	1,97E-02
SAOUHSC_02971	*aur*	Zinc metalloproteinase aureolysin	142,26	421,39	-2,99	0,978038022	2,20E-02
SAOUHSC_00435	*gltB*	Glutamate synthase large subunit	26,27	87,09	-3,37	0,966534151	3,35E-02
SAOUHSC_01788	*thrS*	Threonyl-tRNA synthetase	198,20	365,34	-1,85	0,965433999	3,46E-02
SAOUHSC_00987	*sspB*	Cysteine protease	153,57	463,53	-3,06	0,96542764	3,46E-02
SAOUHSC_00248	*lytM*	Peptidoglycan hydrolase	66,97	305,62	-4,49	0,965036005	3,50E-02
SAOUHSC_02571	*ssaA*	Secretory antigen	138,98	338,24	-2,48	0,964991889	3,50E-02
SAOUHSC_00427	*sle1*	Autolysin	429,09	1055,24	-2,42	0,964980764	3,50E-02
SAOUHSC_02941	*nrdG*	Hypothetical	1227,38	2587,23	-2,13	0,964320831	3,57E-02
SAOUHSC_01001	*qoxA*	Quinol oxidase subunit I	697,44	1145,04	-1,67	0,964256188	3,57E-02
SAOUHSC_00964		Hypothetical	556,54	1940,78	-3,40	0,963480675	3,65E-02
SAOUHSC_00401		Hypothetical	1403,05	3417,37	-2,46	0,963157589	3,68E-02
SAOUHSC_00717	*saeP*	Hypothetical	454,09	1428,65	-3,19	0,963014533	3,70E-02
SAOUHSC_00741	*nrdI*	Ribonucleotide reductase stimulatory protein	126,39	380,26	-2,99	0,962644542	3,74E-02
SAOUHSC_01942	*splA*	Serine protease SplA	105,54	359,35	-3,44	0,962415601	3,76E-02
SAOUHSC_00083	*sbnI*	Hypothetical	363,87	881,28	-2,47	0,962365372	3,76E-02
SAOUHSC_00348	*rpsF*	30S ribosomal protein S6	982,60	1832,88	-1,87	0,962209199	3,78E-02
SAOUHSC_00436	*gltD*	Glutamate synthase subunit beta	28,31	115,63	-4,17	0,962088791	3,79E-02
SAOUHSC_00051	*plc*	1-phosphatidylinositol phosphodiesterase	202,58	448,04	-2,24	0,961868924	3,81E-02
SAOUHSC_00411.2	*psma2*	Alpha phenol soluble modulin	6294,38	16245,15	-2,65	0,961851107	3,81E-02
SAOUHSC_01121	*hla*	Alpha-hemolysin	706,11	1447,83	-2,10	0,961834859	3,82E-02
SAOUHSC_00272		Hypothetical	184,04	859,78	-4,58	0,961776566	3,82E-02
SAOUHSC_00801	*secG*	Preprotein translocase subunit SecG	584,74	1362,06	-2,34	0,961539688	3,85E-02
SAOUHSC_01935	*splF*	Serine protease SplF	5265,82	14061,34	-2,77	0,961147762	3,89E-02
SAOUHSC_02369	*rpoE*	DNA-directed RNA	172,87	497,08	-2,87	0,960776584	3,92E-02

		polymerase subunit delta					
SAOUHSC_00268		Hypothetical	45,12	113,29	-2,56	0,960395256	3,96E-02
SAOUHSC_01110	*efb*	Fibrinogen-binding protein-like protein	126,27	250,44	-1,99	0,960278817	3,97E-02
SAOUHSC_01688	*lepA*	GTP-binding protein LepA	39,12	92,87	-2,38	0,960050254	3,99E-02
SAOUHSC_02855	*amiD2*	LysM domain-containing protein	358,40	782,76	-2,18	0,960029998	4,00E-02
SAOUHSC_02762		Hypothetical	918,78	1826,28	-1,99	0,960008689	4,00E-02
SAOUHSC_02114	*dagK*	Putative lipid kinase	90,73	194,18	-2,15	0,960006118	4,00E-02
SAOUHSC_02372		Hypothetical	137,84	307,63	-2,28	0,95994154	4,01E-02
SAOUHSC_02430	*htsA*	ABC transporter periplasmic binding protein	274,46	492,42	-1,82	0,958977681	4,10E-02
SAOUHSC_01320	*dhoM*	Homoserine dehydrogenase	362,47	704,97	-1,99	0,958905848	4,11E-02
SAOUHSC_00986	*sspC*	Cysteine protease	451,73	1107,05	-2,49	0,958676652	4,13E-02
SAOUHSC_00411.3	*psma3*	Alpha phenol soluble modulin	110,16	301,80	-2,77	0,958605079	4,14E-02
SAOUHSC_02112		Hypothetical	186,12	519,73	-2,80	0,957470594	4,25E-02
SAOUHSC_02972	*isaB*	Immunodominant antigen B	677,00	1537,03	-2,23	0,956063948	4,39E-02
SAOUHSC_02885		Hypothetical	132,07	246,86	-1,89	0,955669456	4,43E-02
SAOUHSC_01326	*lysP2*	Hypothetical	99,33	202,49	-2,04	0,955660583	4,43E-02
SAOUHSC_02127	*sspB2*	Staphopain thiol proteinase	43,14	172,21	-4,09	0,955630096	4,44E-02
SAOUHSC_01936	*splE*	Serine protease SplE	87,44	249,28	-2,90	0,955621944	4,44E-02
SAOUHSC_00728	*ltaS*	Hypothetical	272,82	431,26	-1,60	0,955543211	4,45E-02
SAOUHSC_00625	*mnhA*	Putative monovalent cation/H+ antiporter subunit A	109,46	262,11	-2,44	0,955398071	4,46E-02
SAOUHSC_02763	*opp-1F*	Peptide ABC transporter ATP-binding protein	121,15	342,65	-2,89	0,955047715	4,50E-02
SAOUHSC_00988	*sspA*	Glutamyl endopeptidase	82,82	281,94	-3,41	0,95493893	4,51E-02
SAOUHSC_00711		Hypothetical	108,83	207,04	-1,93	0,95476044	4,52E-02
SAOUHSC_00561	*vraX*	Hypothetical	18270,26	34742,56	-1,90	0,954606095	4,54E-02
SAOUHSC_02550	*FdhD*	Formate dehydrogenase accessory protein	158,68	349,10	-2,20	0,954144948	4,59E-02
SAOUHSC_00875	*ndh2*	Hypothetical	75,00	161,09	-2,17	0,953936808	4,61E-02
SAOUHSC_01359	*mprF*	Hypothetical	97,32	172,27	-1,77	0,953927083	4,61E-02
SAOUHSC_01192	*vfrA*	Hypothetical	80,35	283,11	-3,54	0,95391581	4,61E-02
SAOUHSC_02887	*isaA*	Immunodominant antigen A	2932,90	4256,92	-1,47	0,953569593	4,64E-02
SAOUHSC_02254	*groEL*	chaperonin GroEL	116,75	289,12	-2,52	0,953545852	4,65E-02

SAOUHSC_02485	*rpoA*	DNA-directed RNA polymerase subunit alpha	315,26	1489,48	-4,90	0,953422764	4,66E-02
SAOUHSC_01462	*gpsB*	Hypothetical	243,57	577,38	-2,40	0,953097863	4,69E-02
SAOUHSC_00367	*tcyP*	Hypothetical	66,01	234,27	-3,65	0,953085711	4,69E-02
SAOUHSC_01062		Hypothetical	763,80	1671,30	-2,21	0,952927742	4,71E-02
SAOUHSC_00893	*namA*	FMN oxidoreductase	217,11	427,79	-1,96	0,952418385	4,76E-02
SAOUHSC_00144	*ausA*	Hypothetical	75,27	132,50	-1,81	0,952097951	4,79E-02
SAOUHSC_00652	*fhuA*	Iron compound ABC transporter ATP-binding protein	208,31	411,63	-1,99	0,952073482	4,79E-02
SAOUHSC_02883	*ssaA*	LysM domain-containing protein	98,31	253,33	-2,56	0,951724142	4,83E-02
SAOUHSC_01431	*msrB*	Methionine sulfoxide reductase B	240,56	441,65	-1,85	0,950957464	4,90E-02

[a] Transcripts Per Kilobase Million; [b] Probability of differential expression; [c] False discovery rate

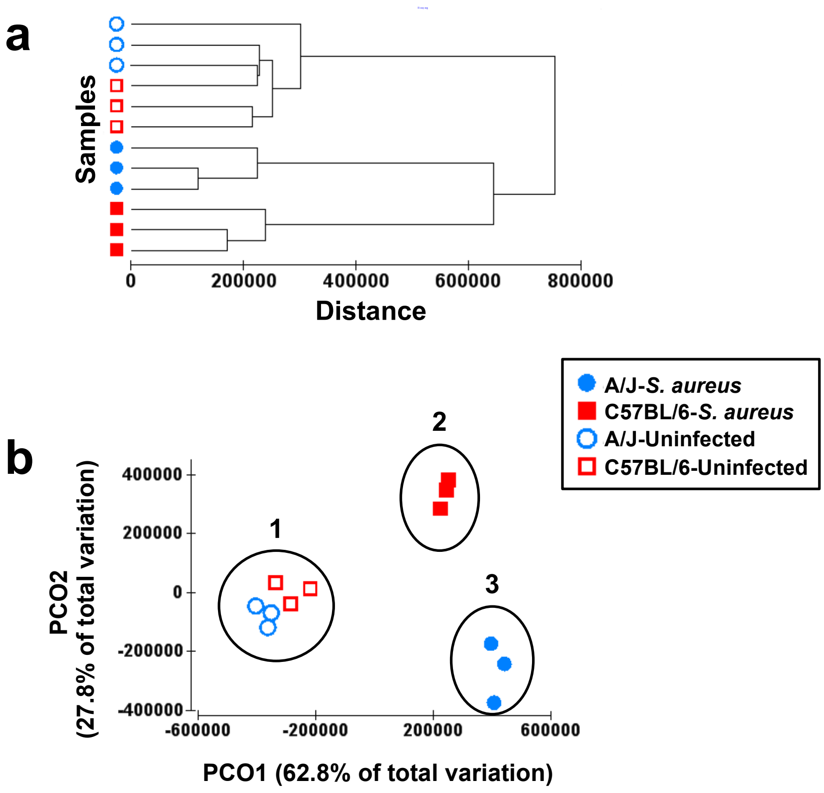

Supplementary Figure 1. Global analysis of gene expression in *S. aureus*-infected versus uninfected A/J and C57BL/6 mice. (**a**) Hierarchical dendogram showing the clustering of gene expression between tissue samples from uninfected and *S. aureus*-infected A/J and C57BL/6 mice. (**b**) PCA analysis of the above mentioned transcriptome datasets. Circles are drawn around group of samples clustering together.

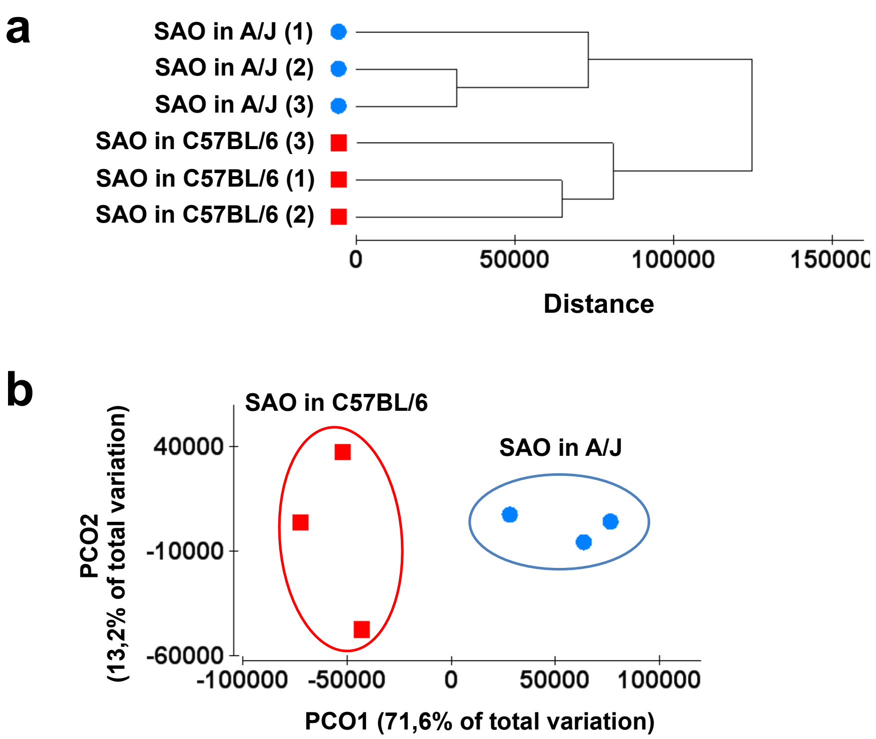

Supplementary Figure 2. Global analysis of *S. aureus* gene expression during infection of A/J and C57BL/6 mice. (a) Hierarchical dendogram showing the clustering of S. aureus gene expression in A/J (SAO in A/J) and C57BL/6 (SAO in C57BL/6) mice. **(b)** PCA analysis of the above mentioned transcriptome datasets. Circles are drawn around group of samples clustering together.

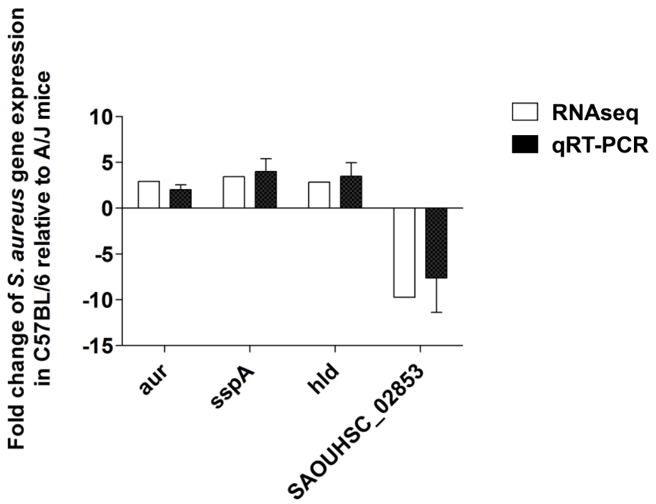

b

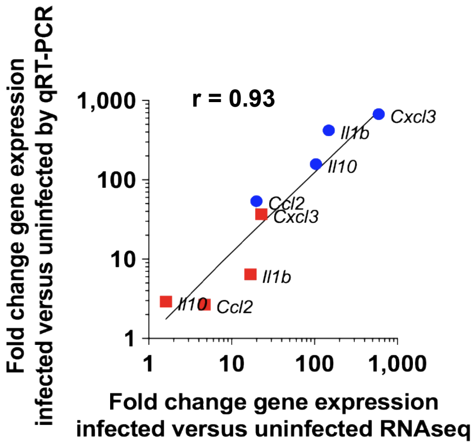

Supplementary Figure 3. Validation of dual RNA-seq data using qRT-PCR. (a) Bacterial RNA-seq data were validated by comparing the fold change of a selected set of genes by *S. aureus* during infection of C57BL/6 mice versus A/J mice as determined by NOISeq analysis of the RNA-seq data (white bars) with the fold change of the same genes calculated by qRT-PCR (black bars). Each bar represents the mean value of three independent experiments. **(b)** Host RNA-seq data were validated by comparing the fold change in gene expression of a selected set of genes in the kidneys of *S. aureus*-infected versus uninfected A/J (blue circles) and C57BL/6 (red squares) mice as determined by DESeq2 analysis of RNA-seq data (x-axis) with the fold change of those genes calculated by qRT-PCR (y-axis). The Pearson correlation coefficient r between the two methods is shown.

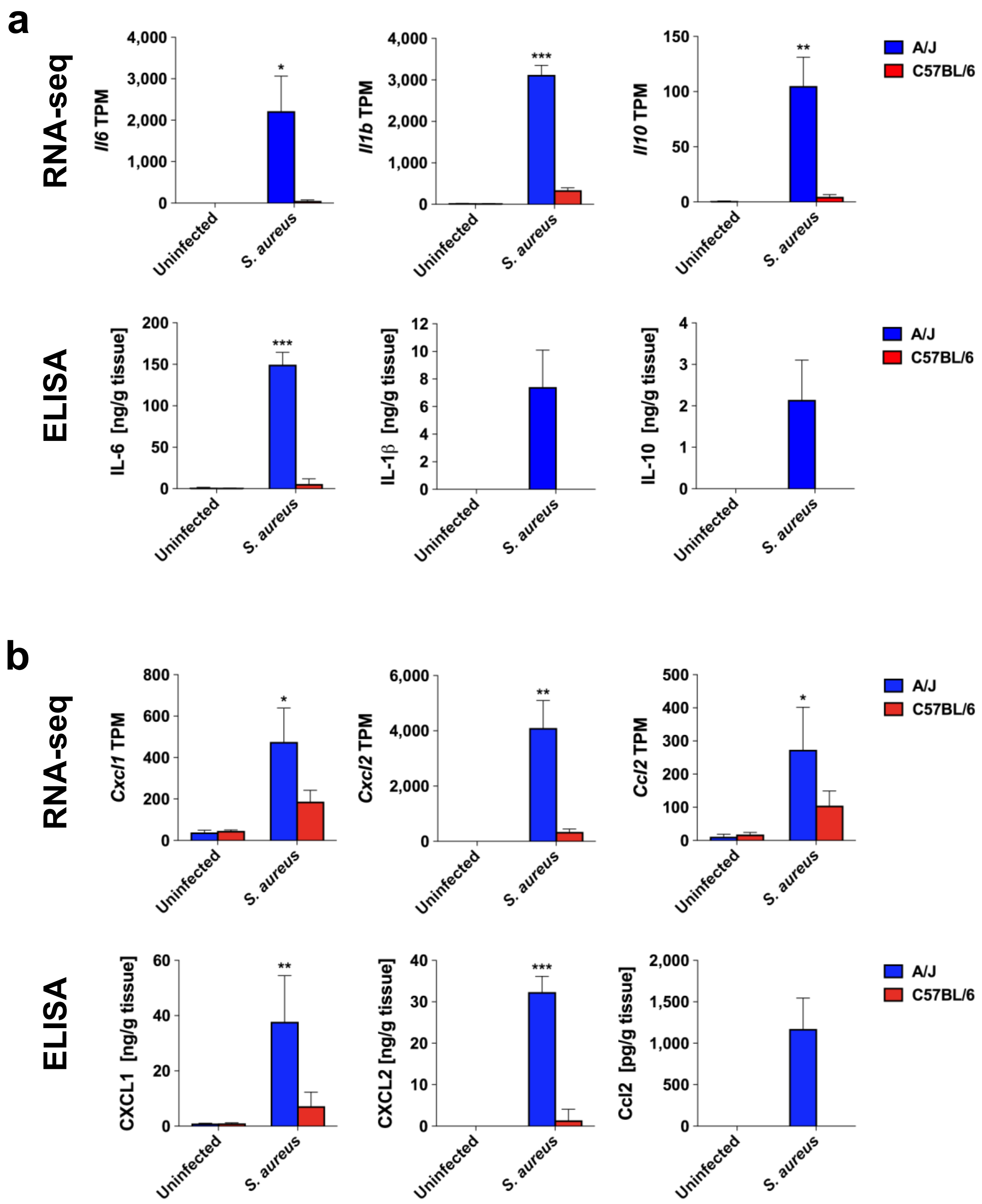

Supplementary Figure 4. Validation of a RNA-seq dataset at the protein level using ELISA. (a) Cytokine and **(b)** chemokine levels of mRNA and proteins in kidney tissue from uninfected controls and *S. aureus*-infected A/J (blue bars) and C57BL/6 (red bars) mice. The mRNA and protein levels in the infected tissue were statistically compared between infected A/J and C57BL/6 mice (n= 3, *t*-test, * $p < 0.05$, ** $p < 0.01$, *** $p < 0.001$).
